# Biodiversity research in the “big data” era: *GigaScience* and Pensoft work together to publish the most data-rich species description

**DOI:** 10.1186/2047-217X-2-14

**Published:** 2013-10-28

**Authors:** Scott C Edmunds, Chris I Hunter, Vincent Smith, Pavel Stoev, Lyubomir Penev

**Affiliations:** 1 GigaScience, BGI HK Ltd., Tai Po, Hong Kong; 2 The Natural History Museum, Cromwell Road, London, UK; 3 Pensoft Publishers, Sofia, Bulgaria; 4 National Museum of Natural History Museum, Sofia, Bulgaria; 5 Institute for Biodiversity and Ecosystem Research, Bulgarian Academy of Sciences, Sofia, Bulgaria

## Abstract

With the publication of the first eukaryotic species description, combining transcriptomic, DNA barcoding, and micro-CT imaging data, *GigaScience* and Pensoft demonstrate how classical taxonomic description of a new species can be enhanced by applying new generation molecular methods, and novel computing and imaging technologies. This 'holistic’ approach in taxonomic description of a new species of cave-dwelling centipede is published in the *Biodiversity Data Journal* (*BDJ*), with coordinated data release in the *GigaScience* GigaDB database.

## Background

### The challenge

While much has been written on the data deluge in genomics, biodiversity research has undergone a similar explosion in the throughput and volume of data produced. With increasingly threatened habitats, free and open access to this data is essential for informed decision-making on conservation issues. Much of this growth has been led by advances in DNA barcoding, and by combining bulk-sampling with genomic technology, the technique of metabarcoding will increase this flood of data even further. With growing intensities in sampling via mass sampling of arthropods, mass detection of environmental DNA in aquatic environments, and broad overviews of plant communities, these sophisticated analyses allow temporal and spatial assessment of biodiversity across varied environments at previously unobtainable levels of detail.

These new ecoinformatics and biomonitoring techniques are able to work quantitively [1], so in addition to ecosystem assessment, they also allow biodiversity surveys and the discovery of new species, even inside metropolitan areas that should be comparatively well sampled [1].

Traditional descriptive taxonomy has failed to keep pace with the explosive growth of sequencing. As a consequence there has been a huge increase in the number of “dark taxa” within public sequence databases. These are taxa that are not identified to a known species, either because they are new to science, or because the specimen has never been identified. In many cases dark taxa are already represented within museum collections and have published descriptions However, there is no mechanism by which taxonomists can easily verify the identity of dark taxa, and even if there were, describing them quickly and efficiently was impossible until recently, due to the nomenclatural rules prohibiting the description of new species in electronic only publications. The increasing pace of species extinction, coupled with the decreasing pool of taxonomic expertise, means that there is an urgent need to speed up the process of investigating biodiversity.

### Potential solutions

From September 2012 the process of describing animal species joined the electronic era, with the acceptance of electronic taxonomy publication and registration with ZooBank, the official registry of the ICZN (International Trust for Zoological Nomenclature). The genomic explosion has led to a rapid increase in the number of reference genomes, and the production of transcriptomes is becoming an even faster and more cost-effective substitute to produce massive amounts of gene sequence data for genetic and phylogenomic studies. The pace of traditional taxonomy is, in some instances, catching up with genome sequencing, as was demonstrated with a new Strepsiptera genome [2] which was published back-to-back with its species description in *Zookeys*[3].

While the barcoding community has produced workarounds for the lack of species descriptions, such as the use of interim taxonomic nomenclature (operational taxonomic units) in their sample registries, the use of, DNA-based classifications were initially restricted to 'taxonomy-free’ groups such as bacteria and fungi. The new Barcode Index Number (BIN) system allows clustering of sequences into "BINs", and can aid revisionary taxonomy by flagging possible cases of synonymy [4].

On top of advances in sequencing technology, new imaging techniques are providing ways to study morphology and animal behavior in unprecedented and reproducible detail, and in a non-destructive manner. Subrobotic digital imaging can rapidly process stacks of images through collections. Digital video allows for archiving of *in-situ* behavior, while the use of X-ray micro-computed tomography scanning (microCT) supports three-dimensional virtual representations of materials. The use of these data as virtual type specimens has been promoted through the concept of “cybertypes”. These digital representations of exemplar specimens create the potential for new forms of collections that can be openly accessed and used without the physical constraining of loaning specimens or visiting natural history collections [5].

Some have suggested a 'turbo-taxonomy’ approach, combining all of these techniques to address a perceived decline in taxonomic expertise [6,7]. This putative pipeline has recently been demonstrated with large series of parasitic wasps [6] and *Trigonopterus* weevils [7]. While these examples have focused on taxonomic throughput, less attention has been given to the potential to integrate these different data types.

### The example

*GigaScience* and Pensoft Publishers present the results of a pilot study aiming to demonstrate how the classical taxonomic description of a new species can be enhanced by utilizing the latest molecular methods, and novel computing and imaging technologies. A new species of cave-dwelling centipede, *Eupolybothrus cavernicolus* Komerički & Stoev (Chilopoda: Lithobiomorpha: Lithobiidae) [8], recently discovered underground in a Croatian cave, is the first Eukaryotic species description for which, in addition to traditional morphological description, the authors provide a fully sequenced transcriptome, DNA barcodes and BIN entries, detailed anatomical X-ray micro-CT scans, as well as a movie of the living specimen to document important traits of its behavior [9].

Communicating the results of next generation sequencing effectively requires the next generation of data publishing. The description published in the newly launched *Biodiversity Data Journal* (*BDJ*) aims to provide a gold standard for not just the quantity and diversity of data available, but for quality and amount of metadata to make this data reusable and interoperable. It also demonstrates the benefits of integrating a scholarly publishing workflow that allows authors, curators and editors to write, peer-review, publish, and disseminate biodiversity data within a single web-based platform [10]. *GigaScience*’s contribution to the pilot is using the GigaDB database for large-scale data handling, management, curation and storage (see [9]). The data are also available in relevant community specific databases, with transcriptomic sequencing data in both ENA and ArrayExpress, plus annotation data made publically available through ArrayExpress to the most stringent (MINSEQE) metadata standards. Imaging data is deposited in morphological databases, and biodiversity data in the Barcode of Life databases. All data are made available with no restrictions on reuse under the most open CC0 public domain waiver. The publication of Stoev et al., in this manner provides a significant step forward from integrating small data sets in the article text in both computer- and human-readable formats, into the world of big data publishing.

To tackle complex and novel scientific questions, datasets and metadata from different sources need to be harmonized and made interoperable. Working with the ISA community we have provided metadata in the interoperable ISA-TAB format to maximize the discovery, exchange and informed integration of these diverse datasets. Until recently there has been a lack of incentives for data producers to make their data available, but this data note provides an example of how credit can be obtained for providing this effort. While the focus is on providing data rather than analysis, there are interesting questions to be asked such as on the evolution of the species, development of its segmented body structure, and how it has adapted to its dark cave environment. By providing such a diverse range of phenotypic and molecular data in an integrated and reusable form, we hope to enable other researchers to explore these and other questions. While this new species subterranean lifestyle could hopefully protect it from some of the growing threats surface habitats are encountering, this new type of species description also provides an example of how much previously uncharacterized information on its behavior, internal structure, physiology and genetic make-up can be preserved for future generations.

## Abbreviations

BDJ: Biodiversity data journal; BIN: Barcode index number; ICZN: International trust for zoological nomenclature; micro-CT: micro-computed tomography.

## Competing interests

SCE and CIH are employed by *GigaScience* and BGI Hong Kong. VS is Editor-in-Chief of *BDJ*. PS and LP are employed by Pensoft.

## Authors’ contributions

The authors wrote the editorial based on the editorial policies of *GigaScience* and *BDJ*.

## References

[B1] Zhou X Li Y Liu S Yang Q Su X Zhou L Huang Q Ultra-deep sequencing enables high-fidelity recovery of biodiversity for bulk arthropod samples without PCR amplification GigaScience 2013 2 1 4 doi:10.1186/2047-217X-2-4 10.1186/2047-217X-2-4 23587339 PMC3637469

[B2] Niehuis O Hartig G Grath S Pohl H Lehmann J Tafer H Misof B Genomic and morphological evidence converge to resolve the enigma of strepsiptera Curr Biol 2012 Retrieved from http://www.sciencedirect.com/science/article/pii/S0960982212005714 10.1016/j.cub.2012.05.018 22704986

[B3] Pohl H Niehuis O Gloyna K Misof B Beutel RG A new species of Mengenilla (Insecta, Strepsiptera) from Tunisia ZooKeys 2012 2 198 79 101 doi:10.3897/zookeys.198.2334 22707907 10.3897/zookeys.198.2334 PMC3368257

[B4] Ratnasingham S Hebert PDN A DNA-based registry for all animal species: the barcode index number (BIN) system. (D. Fontaneto, Ed.) PLoS One 2013 2 7 e66213 doi:10.1371/journal.pone.0066213 10.1371/journal.pone.0066213 23861743 PMC3704603

[B5] Faulwetter S Vasileiadou A Kouratoras M Dailianis T Arvanitidis C Micro-computed tomography: Introducing new dimensions to taxonomy ZooKeys 2013 2 263 1 45 doi:10.3897/zookeys.263.4261 23653515 10.3897/zookeys.263.4261 PMC3591762

[B6] Butcher BA Smith MA Sharkey MJ Quicke Donald LJ A turbo-taxonomic study of Thai Aleiodes (Aleiodes) and Aleiodes (Arcaleiodes) (Hymenoptera: Braconidae: Rogadinae) based largely on COI barcoded specimens, with rapid descriptions of 179 new species Zootaxa 2012 2 1 232 Retrieved from http://www.mapress.com/zootaxa/list/2012/3457.html

[B7] Riedel A Sagata K Suhardjono YR Tänzler R Balke M Integrative taxonomy on the fast track - towards more sustainability in biodiversity research Front Zool 2013 2 1 15 doi: 10.1186/1742-9994-10-15 10.1186/1742-9994-10-15 23537182 PMC3626550

[B8] Stoev P Komerički A Akkari N Liu S Zhou X Weigand AM Hostens J Hunter CI Edmunds SC Porco D Zapparoli M Georgiev T Mietchen D Roberts D Smith V Penev L *Eupolybothrus cavernicolus* Komerički & Stoev, sp. n. (Chilipoda: Lithobiomorpha: Lithobiidae): The first Eukaryotic species description combining transcriptomic, DNA barcoding, and micro-CT imaging data Biodivers Data J 2013 2 e1013 doi:10.3897/BDJ.1.e1013 24723752 10.3897/BDJ.1.e1013 PMC3964625

[B9] Stoev P Komerički A Akkari N Liu S Zhou X Weigand AM Hostens J Porco D Penev L Transcriptomic, DNA barcoding, and micro-CT imaging data from an advanced taxonomic description of a novel centipede species (*Eupolybothrus cavernicolus* Komerički & Stoev, sp. n.) Gigascience Database 2013 http://dx.doi.org/10.5524/100063

[B10] Smith V Georgiev T Stoev P Biserkov J Miller J Livermore L Penev L Beyond dead trees: integrating the scientific process in the Biodiversity Data Journal Biodivers Data J 2013 2 e995 doi:10.3897/BDJ.1.e995 10.3897/BDJ.1.e995 24723782 PMC3964700

